# Viral Tropism

**DOI:** 10.3389/fmicb.2012.00281

**Published:** 2012-08-03

**Authors:** Masako Nomaguchi, Mikako Fujita, Yasuyuki Miyazaki, Akio Adachi

**Affiliations:** ^1^Department of Microbiology, Institute of Health Biosciences, The University of Tokushima Graduate SchoolTokushima, Japan; ^2^Research Institute for Drug Discovery, School of Pharmacy, Kumamoto UniversityKumamoto, Japan

One of the most important and outstanding characteristics of viruses is their cellular and host tropism (Levine and Enquist, 2007). As parasitic entities, viruses have to compromise with numbers of positive and negative factors present in target cells for their survival. In the absence of an appropriate interaction with cells, they do not replicate at all. Viral tropism therefore can be determined at each replication step, beginning with the entry into cells and ending with the progeny production from cells. There are two major types of viral tropism, that is, the receptor-dependent and -independent tropisms. Restriction of viral replication occurs on the cell surface (receptor-dependent viral entry step) and/or intracellularly (receptor-independent post-entry replication steps).

In this Research Topic, a number of basic studies on both types of animal virus tropism have been published as either reviews, mini-reviews, or an original research article. Kajitani et al. (2012) have efficiently summarized a unique and complex lifecycle of human papilloma viruses (double-stranded DNA virus). Ohka et al. (2012) have concisely described the receptor-dependent and -independent tropism of poliovirus (positive sense single-stranded RNA virus). Ramadhany et al. (2012) have presented an original study on the mutations in hemagglutinin gene of influenza virus (negative sense single-stranded and segmented RNA virus) and have discussed their effects on the viral tropism. Multiple cellular receptors for measles virus (negative sense single-stranded and segmented RNA virus) have been clearly described by Sato et al. (2012). Takada (2012) has proposed models of filovirus (ebola and Marburg viruses; negative sense single-stranded RNA virus) entry into cells. Yasuda (2012) has discussed the interaction of *ebolavirus* and anti-viral cellular factor tetherin/BST-2. Species tropism of human and simian immunodeficiency viruses (HIV and SIVs; positive sense single-stranded RNA viruses containing reverse transcriptase), a representative of the receptor-independent tropism, has been systematically summarized by Nakayama and Shioda (2012). Sakuma and Takeuchi (2012) has provided insight into the mechanism for species-specific SIV replication. Nomaguchi et al. (2012) have focused on the function of viral accessory proteins, and discussed the bases for HIV-1 species tropism. Tani et al. (2011) have described the use of vesicular stomatitis virus (negative sense single-stranded RNA virus) as tools in various research and medical fields. Finally, Uchiyama (2012) has reviewed the rickettsia tropism as an example of non-viral microbes (obligate intracellular parasitic bacterium).

The authors in this Research Topic have highlighted the importance of viral tropism by presenting biological phenomena and their underpinning host cellular and molecular bases. The biologically unique phenomenon of viral tropism is not only of interest to virologists to bring progress to their basic science but also highly relevant to clinical studies with the goal to design new anti-viral strategies. Accordingly, “Viral Tropism” must be considered a sibling to another Research Topic in Frontiers in Virology entitled “Receptor usage and pathogenesis in acute and chronic viral infection.”
